# Photoreceptor rescue of pigment epithelium-derived factor-impregnated nanoparticles in Royal College of Surgeons rats

**Published:** 2012-12-29

**Authors:** Goichi Akiyama, Tsutomu Sakai, Noriyuki Kuno, Erika Kimura, Kiichiro Okano, Hideo Kohno, Hiroshi Tsuneoka

**Affiliations:** 1 Department of Ophthalmology, Jikei University School of Medicine, Jikei University School of Medicine, Tokyo, Japan; 2 Research and Development Center, Santen Pharmaceutical Co., Ltd, Nara, Japan

## Abstract

**Purpose:**

To investigate the protective effect of intravitreal injection of pigment epithelium-derived factor-impregnated nanoparticles (PEDF-NPs) against photoreceptor degeneration in Royal College of Surgeons (RCS) rats.

**Methods:**

Three-week-old RCS rats received an intravitreal injection of PBS, blank NPs, PEDF (2.5 μg), or PEDF-NPs (2.5 μg). Eyes were assessed with morphological, immunohistochemical, and physiologic analysis over the following 8 weeks. Cell death was examined using the terminal deoxynucleotidyl transferase-mediated uridine 5′-triphosphate-biotin nick end labeling (TUNEL) assay.

**Results:**

In RCS rats, the a- and b-wave amplitudes on electroretinograms in eyes treated with PEDF-NPs were greater than those in retinas receiving other treatment. Immunocytochemistry showed consistently greater opsin preservation in eyes treated with PEDF-NPs. A significantly higher number of photoreceptors and significantly fewer TUNEL-positive cells were present after treatment with PEDF-NPs, compared to PEDF-treated eyes.

**Conclusions:**

The results suggest that intravitreally injected PEDF-NPs delayed photoreceptor degeneration by inhibiting apoptosis in the RCS rat retina due to targeting and sustained release of PEDF.

## Introduction

Pigment epithelium-derived factor (PEDF) has a protective neurotrophic activity in mice and rats with photoreceptor degeneration [1,2]. A single intravitreal or subretinal injection of PEDF delays photoreceptor degeneration, but such treatment does not allow long-term rescue of photoreceptors due to the short half-life of the trophic factor. Therefore, recent “photoreceptor rescue” studies using PEDF have focused on efficient approaches for continuous delivery; for example, gene therapy with viral vectors has been found to be useful for preventing the progression of photoreceptor degeneration in rats [3,4]. The prolonged effect of PEDF is presumably beneficial; however, several problems remain to be solved for potential human applications. In particular, some viral vectors are biologically unsafe due to severe immune reactions or oncogenic effects, and subretinal injection for PEDF gene transfer has potential risks for retinal detachment and proliferative vitreoretinopathy. Thus, developing a delivery system that permits enhanced localization of PEDF at the target site and sustained drug release without causing serious complications is important.

In a previous study, we described the efficient preparation of gelatin nanoparticles (NPs) with fibroblast growth factor (FGF), and showed that intravitreal injection of NPs with FGF (FGF-NPs) prolonged the photoreceptor protective effect of FGF due to efficient targeting and sustained drug retention in Royal College of Surgeons (RCS) rats [5]. FGF-NPs exhibited significant photoreceptor protection, but the repeated injections induced cataract formation and retinal gliosis in some cases. Here, we selected PEDF for conjugation to NPs to avoid these serious adverse effects. Our hypothesis was that intravitreal injection of PEDF-NPs would increase the efficacy of PEDF in preventing photoreceptor degeneration through improved targeting and sustained release. In this study, we examined the protective effect of intravitreally injected PEDF-NPs against photoreceptor degeneration in RCS rats.

## Methods

### Animals and anesthesia

RCS rats obtained from CLEA Japan (Tokyo, Japan) were used in the study. All experiments were conducted in accordance with the Association for Research in Vision and Ophthalmology Statement for the Use of Animals in Ophthalmic and Vision Research. The rats were anesthetized with a mixture (1:1) of ketamine hydrochloride (10 mg/kg; Wako Pure Chemicals Industries, Ltd., Osaka, Japan) and xylazine hydrochloride (4 mg/kg; Wako).

### Preparation of pigment epithelium-derived factor-impregnated nanoparticles

PEDF-NPs were prepared as follows, using basic gelatin isolated from bovine bone collagen by an alkaline process (AP-150, Nitta Gelatin, Osaka, Japan; isoelectric point: 9.0, molecular weight: 100,000) and human recombinant PEDF (Upstate, Temecula, CA). Cross-linked gelatin NPs were prepared through a dehydrothermal process and ultraviolet irradiation of preprepared non-crosslinked gelatin particles. The details have been reported previously [5]. In brief, gelatin aqueous solution (100 mg/ml, 0.2 ml) was added to pre-heated olive oil (5ml) at 40 °C, followed by agitation for 1 min. The resulting emulsion was cooled in crushed ice to allow solidification of aqueous gelatin droplets. Acetone was then added to the emulsion and stirring was continued for 1h at 4 °C. The resulting particles were washed three times with acetone and recovered by centrifugation at 3500 rpm and 4 °C for 5 min. The non-crosslinked gelatin particles were dried in a dessiccator at 4 °C and then placed in a glass dish and heated at 160 °C for 72 h. The gelatin particles were then exposed to ultraviolet irradiation for 30 min, after which the particles were suspended in distilled water and filtrated using a membrane filter (pore size: 1.0 µm). The filtrate was freeze-dried and cross-linked NPs were obtained. The NP diameter was measured with dynamic light scattering (NICOMP-370, Particle Sizing Systems, Santa Barbara, CA), and the average diameter was approximately 585 nm.

PEDF was incorporated into the gelatin NPs by dropping 5 mg/ml of PEDF solution (20 µl) onto 2 mg of freeze-dried gelatin NPs, which were then left to stand at 4 °C for 12 h. The solution (20 µl) was completely absorbed into the NPs during swelling, since the solution volume was less than that required theoretically for equilibrative swelling of the NPs.

### In vivo evaluation of the intraocular kinetics of pigment epithelium-derived factor

PEDF was radioiodinated using Na^125^I using the chloramine T method. Briefly, 50 μl of an aqueous solution of PEDF (1 mg/ml) was added to a mixture of 10 μl of Na^125^I solution (PerkinElmer, Boston, MA) and 100 μl of chloramine T (Wako) solution (0.2 mg/ml), and incubated for 10 min. Then, 100 μl of sodium metabisulfite (Wako) solution (4 mg/ml) was added to the reaction solution and incubated for 10 min. This final reaction solution was passed through anion-exchange resin (DOWEX 1×8, Dow Chemical Company, Midland, MI). Ten mg of freeze-dried cross-linked gelatin NPs were impregnated with 100 μl of ^125^I-PEDF solution. Five μl of the suspension of ^125^I-labeled PEDF-impregnated NPs were injected into the vitreous of male Wistar rats (n=28, postnatal week 5; Charles River Japan, Yokohama, Japan). At predetermined intervals, rats were sacrificed with sodium pentobarbital (iv; Wako), and eyeballs were enucleated. The remaining radioactivity in the enucleated eyeball was determined with a gamma counter (Cobra II Series Auto-Gamma Counting System, Packard Instrument Co., Meriden, CT).

### Intravitreal injection of pigment epithelium-derived factor-impregnated nanoparticles, pigment epithelium-derived factor, blank nanoparticles, and phosphate buffer solution

Intravitreal injection of the right eyes was performed via the pars plana with 5 µl of PEDF-NPs (containing 2.5 µg PEDF), PEDF (2.5 µg), or blank NPs on P21 (n=15 in each group) in RCS rats, when photoreceptor degeneration was just beginning. The left eyes were uninjected or received phosphate buffered saline (PBS; NaCl 8 g, (Na_2_HPO_4_)12H20 2.9 g, KCl 0.2 g, KH_2_PO_4_ 0.2 g, in H_2_O 1l; pH 7.4; Wako) as the control. The injections were performed with a syringe (80,001; Hamilton Co., Reno, NV) with a 30-gauge needle while viewing the eye under a microscope. Animals with hemorrhage or cataract were excluded.

### Electroretinographic analysis

Scotopic electroretinograms (ERGs) were recorded in both eyes simultaneously with a Ganzfeld bowl. Rats were dark-adapted overnight and prepared under dim red light before being anesthetized with a single intraperitoneal injection of ketamine and xylazine. The pupils were dilated with topical 0.5% tropicamide and 0.5% phenylephrine, and the cornea was anesthetized with topical 0.4% oxybuprocaine hydrochloride.

Gold wire loops were placed on the center of the cornea, reference electrodes were placed subcutaneously under each eye, and a ground electrode was inserted into the tail. ERGs were elicited with 10-ms flashes of white light, and responses were recorded (Synax ER1100; NEC San-ei Instruments, Tokyo, Japan). The a-wave amplitudes were measured from the prestimulus baseline to the bottom of the a-wave, and b-wave amplitudes were measured from the a-wave peak to the most positive peak.

### Histopathology and immunocytochemistry

RCS rats were euthanized with sodium pentobarbital (intravenous; Wako) 4 or 8 weeks after injection (n=4 for each time period), and enucleated eyes were immersion-fixed for 10 min in 4% paraformaldehyde in a sodium cacodylate buffer (0.1 N; pH 7.4; Wako). After the cornea and lens were removed, the eyecup was cut in half. One half of the tissue was stored in the above fixative solution, and small areas of the retina were excised and embedded in low-melting-point agarose (Sigma-Aldrich, St. Louis, MO) for immunocytochemical analysis with confocal microscopy (Laser Scanning System LSM510; Carl Zeiss Meditech, Oberkochen, Germany). The embedded sections were cut on a Vibratome (Leica VT1000S, Leica Microsystems, Heerbrugg, Switzerland) and blocked overnight in normal donkey serum (Jackson Immunoresearch Laboratories Inc., West Grove, PA; 1:20) at 4 °C. The sections were then incubated with primary antibodies overnight at 4 °C on a rotator. The primary antibodies used in this study were a mouse monoclonal antibody (mAb) to rod opsin (Chemicon, Temecula, CA; 1:400), a mouse mAb to vimentin (Sigma; 1:400), a rabbit polyclonal Ab (pAb) to glial fibrillary acidic protein (GFAP, Dako, Glostrup, Denmark; 1:400), a rabbit pAb to medium/long wavelength-sensitive (M/L) cone opsin (Chemicon; 1:200), and a mouse mAb to PEDF (Chemicon; 1:100). All antibody solutions were made in 0.1 M PBS containing 0.5% BSA and 0.1% sodium azide (Sigma-Aldrich; PBTA; 0.1 M PBS containing 0.5% BSA (BSA; Fisher Scientific, Pittsburgh, PA), 0.1% Triton X-100 (Boehringer-Mannheim, Indianapolis, IN), and 0.1% sodium azide [Sigma-Aldrich]). After the sections were rinsed in PBTA, they were incubated with donkey anti-mouse immunoglobulin G (IgG) conjugated to the fluorochrome Cy3 (GFAP, M/L cone opsin), donkey anti-rabbit IgG conjugated to the fluorochrome Cy2 (rod opsin, vimentin), and donkey anti-mouse IgG conjugated to the fluorochrome Cy2 (PEDF; Jackson Immunoresearch Laboratories) overnight at 4 °C on a rotator. The sections were mounted in mounting medium for fluorescence (VECTASHIELD, Vector Laboratories, Inc., Burlingame, CA) and viewed using a laser scanning confocal microscope.

For high-resolution transmitted light microscopy analysis, the other half of the eyecup was immersion-fixed in 1% glutaraldehyde (Wako) and 1% paraformaldehyde (Wako) in a sodium phosphate buffer (0.086 M; pH 7.3) overnight at 4 °C, and then fixed in phosphate-buffered osmium tetroxide (2%; Nisshin-EM Co., Tokyo, Japan) for 1 h and embedded in epoxy resin (Nisshin-EM Co.). The samples were sectioned at 1 µm and stained with toluidine blue (Wako). Morphology was evaluated in the stained retinas.

The number of photoreceptor cells was counted using a standardized approach adapted from a previously described protocol [6]. Three 50-µm sampling bins were established for each of two retinal regions: the superior central and inferior central regions. Three sections were examined for each eye, giving a total of four samples per region and eight samples per eye.

### Terminal deoxynucleotidyl transferase-mediated uridine 5′-triphosphate-biotin nick end labeling assay

Detection of dying (apoptotic) cells was achieved using a terminal deoxynucleotidyl transferase-mediated uridine 5′-triphosphate-biotin nick end labeling (TUNEL) assay, as described previously [7]. The agarose-embedded sections were used in this assay. After the sections were rinsed three times in PBS, they were immersed in 70% alcohol for 30 min, followed by washing in double-distilled water. They were then incubated in 1% citrate and 1% Triton in PBS at 4 °C for 4 min and, after further washing, were then placed in terminal deoxynucleotidyl transferase (TdT) buffer (Boehringer Manheim, Indianapolis, IN) at room temperature for 30 min. They were then incubated with TdT enzyme and 2 µM biotinylated deoxyuridine triphosphate (dUTP; Boehringer Manheim) at 37 °C for 120 min. Finally, the sections were washed in SSC (150 mM sodium chloride and 15 mM sodium citrate, pH 7.4) for 15 min, rinsed with PBS, and examined with a confocal microscope.

TUNEL-positive cells were counted in two areas of each section from four eyes. The percentage of dying photoreceptor cells was expressed as the number of TUNEL-positive cells divided by the total number of nuclei in the outer nuclear layer (ONL). The nuclear counts were normalized using the number of cells/mm of retinal length.

### Statistical analysis

All data are shown as means±standard deviation (SD). Data were analyzed using a non-parametric Mann–Whitney U test. P values less than 0.05 were considered statistically significant.

## Results

### Long-term delivery of ^125^I-labeled pigment epithelium-derived factor-impregnated nanoparticles in rat vitreous

**Figure 1:**
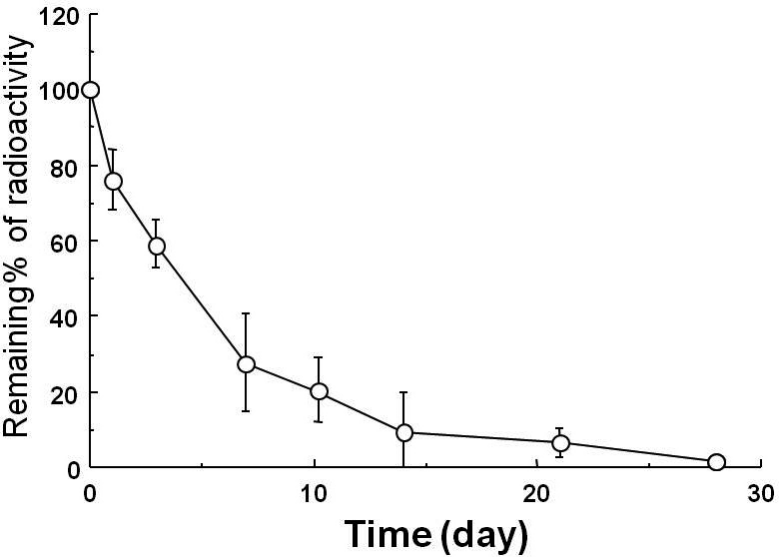
Time course of radioactivity remaining after intravitreal injection of ^125^I-labeled pigment epithelium-derived factor (PEDF)-impregnated nanoparticles in rat vitreous. Five ml of the suspension of ^125^I-labeled PEDF impregnated NPs were injected into the vitreous of male Wistar rats (n=28, postnatal week 5). The remaining radioactivity determined by a gamma counter was 76.1±8, 59.1±6.2, 27.7±13.1, 27.3±8.5, 9.1±10.7, 6.6±4.0, and 1.8±1.0 on Day 1, 3, 7, 10, 14, 21, and 28. Data are shown as the mean±SD (n=4 in each group).

The time course for the remaining radioactivity in rat vitreous after intravitreal injection of ^125^I-labeled PEDF-impregnated NPs is shown in Figure 1. A burst was observed within the first 24 h, and radioactivity from the NPs in the vitreous was sustained over 30 days.

### Effects of pigment epithelium-derived factor-impregnated nanoparticles on morphology

**Figure 2:**
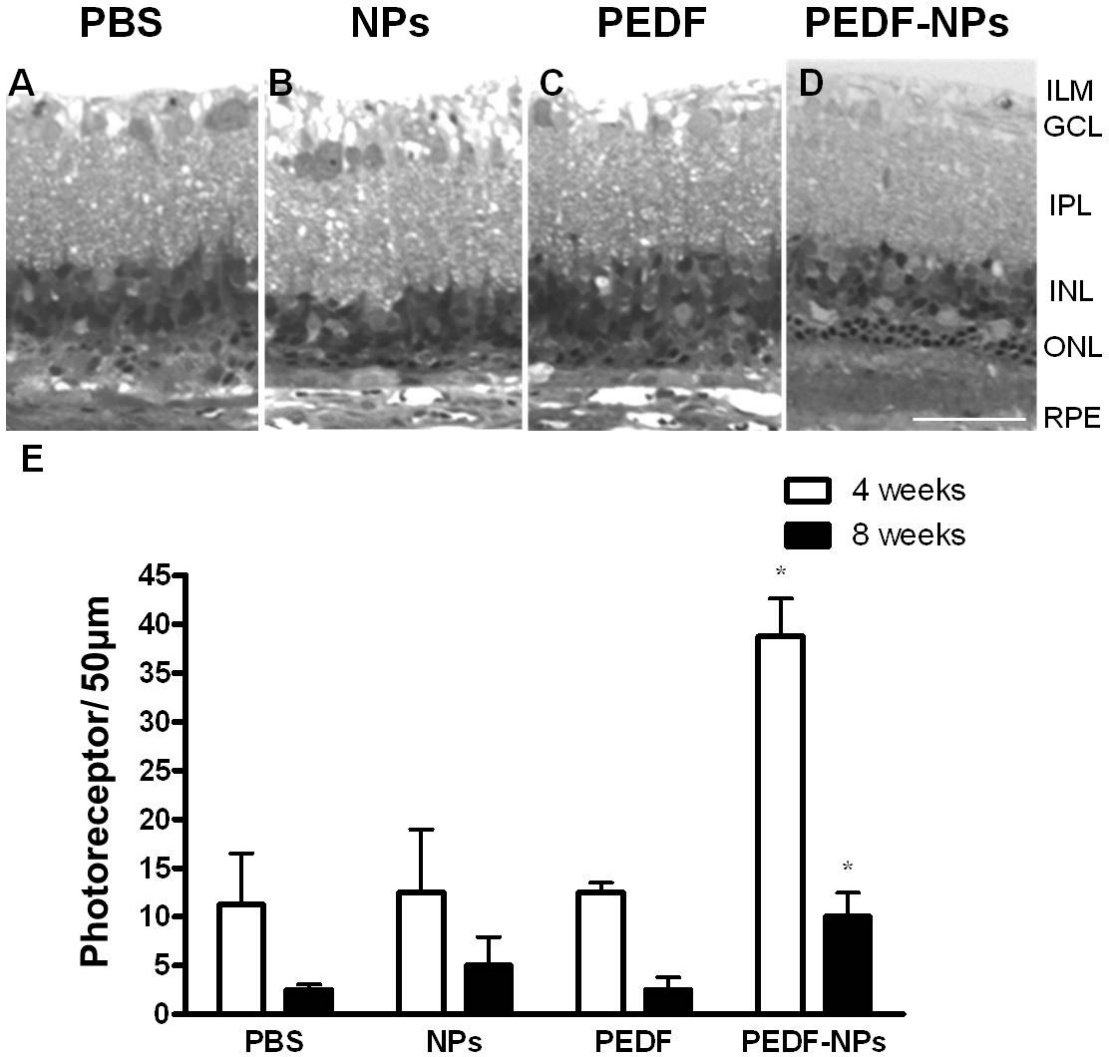
Morphologic rescue of Royal College of Surgeons (RCS) rats. Representative photographs 8 weeks after injection of (**A**) phosphate-buffered saline (PBS), (**B**) blank-nanoparticles (NPs), (**C**) pigment epithelium-derived factor (PEDF), and (**D**) PEDF-NPs. ILM; inner limiting membrane, GCL; ganglion cell layer, IPL; inner plexiform layer, INL; inner nuclear layer, ONL; outer nuclear layer. RPE: retinal pigment epithelium. A bar represents 50 µm. **E**: Mean number of photoreceptors per 50 µm of RCS rat retina at 4 and 8 weeks after injection. Data are shown as the mean±SD (n=4 in each group).

Representative histopathologic features of the superior retina of RCS rats in week 8 following injection are shown in Figure 2A-D. In PBS-, blank NP-, and PEDF-treated eyes, a marked decrease in total nuclei in the ONL was observed (Figure 2A-C), and disruption of the inner and outer segments (OS) of all surviving photoreceptors occurred in all areas. The morphology of eyes treated with PEDF-NPs is illustrated in Figure 2D. In PEDF-NP-treated eyes, the ONL was decreased to two or three rows of photoreceptor nuclei, but PBS-, blank NP-, and PEDF-treated eyes had only a single row of nuclei in the ONL. Thus, the number of surviving photoreceptor nuclei was decreased despite treatment with PEDF-NPs. However, a greater number of photoreceptor nuclei remained in PEDF-NP-treated eyes than in PBS-, blank NP-, and PEDF-treated eyes. The average percentage of remaining nuclei in PEDF-NP-treated eyes was 10.0±2.4%, whereas in PBS-, blank NP-, and PEDF-treated eyes the average percentages were 2.5±0.6%, 5.0±2.9%, and 2.5±1.3%, respectively. The ONL in PEDF-NP-treated eyes was thicker than in PBS-, blank NP-, and PEDF-treated eyes. These features were observed along the entire retina.

The data in Figure 2E (4 and 8 weeks) show the effect of the intravitreal injection of PEDF-NPs on the actual number of nuclei in the ONL, based on cell counts in semithin resin sections. The number of nuclei decreased from 4 to 8 weeks with all treatments. The number of nuclei in the ONL was significantly greater in PEDF-NP-treated eyes relative to eyes treated with PEDF at 4 or 8 weeks (p=0.0172 or p=0.0202; Figure 2E) after injection.

### Immunohistochemical evaluation using confocal microscopy

**Figure 3:**
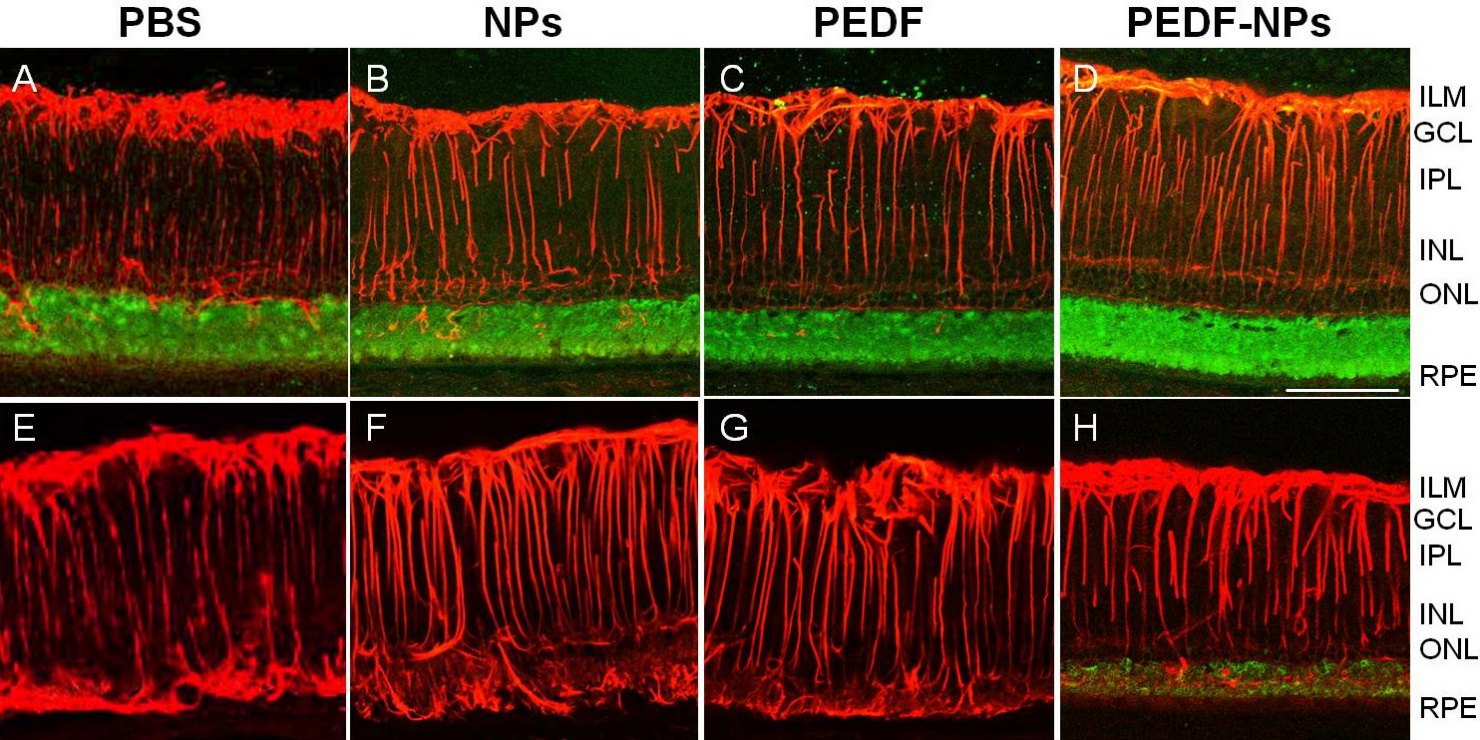
Representative results of phosphate-buffered saline (PBS; **A**, **E**) blank-nanoparticles (NPs; **B**, **F**) pigment epithelium-derived factor (PEDF; **C**, **G**) and PEDF-NP (**D**, **H**) treated eyes double immunostained with antibodies to rod opsin (green) and GFAP (red) at 4 (**A**, **B**, **C**, **D**) and 8 weeks (**E**, **F**, **G**, **H**). ILM; inner limiting membrane, GCL; ganglion cell layer, IPL; inner plexiform layer, INL; inner nuclear layer, ONL; outer nuclear layer. RPE: retinal pigment epithelium. A bar represents 50 µm.

**Figure 4:**
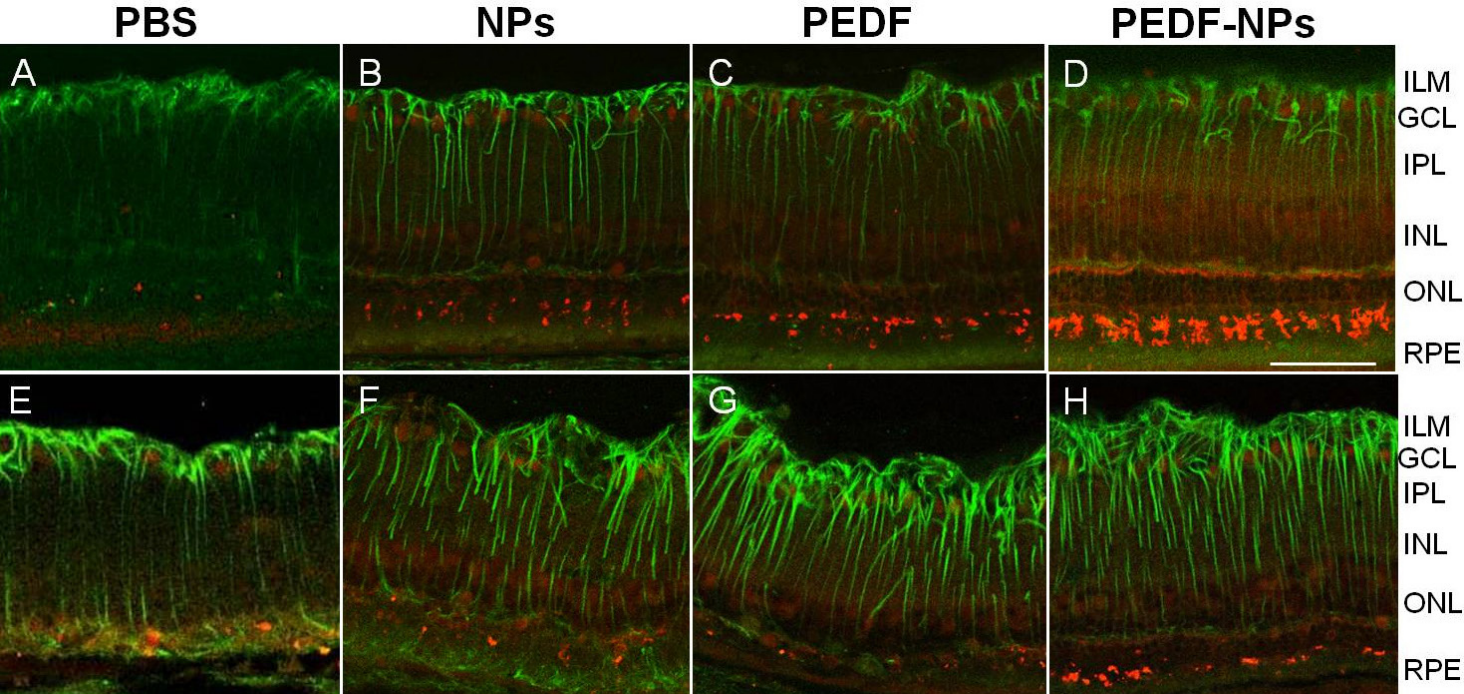
Representative results of phosphate-buffered saline (PBS; **A**, **E**) blank-nanoparticles (NPs; **B**, **F**), pigment epithelium-derived factor (PEDF; **C**, **G**) and PEDF-NP (**D**, **H**) treated eyes double immunostained with antibodies to vimentin (green) and M/L cone opsin (red) at 4 (**A**, **B**, **C**, **D**) and 8 weeks (**E**, **F**, **G**, **H**). ILM; inner limiting membrane, GCL; ganglion cell layer, IPL; inner plexiform layer, INL; inner nuclear layer, ONL; outer nuclear layer. RPE: retinal pigment epithelium. A bar represents 50 µm.

**Figure 5:**
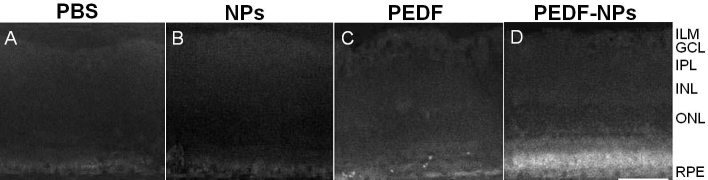
Representative results of phosphate-buffered saline (PBS; **A**), blank-nanoparticles (NPs; **B**) pigment epithelium-derived factor (PEDF; **C**) and PEDF-NP- (**D**) treated eyes immunostained with an antibody to PEDF at 4 weeks. ILM; inner limiting membrane, GCL; ganglion cell layer, IPL; inner plexiform layer, INL; inner nuclear layer, ONL; outer nuclear layer. RPE: retinal pigment epithelium. A bar represents 50 µm.

Immunohistochemical results for retinas collected 4 (A-D) or 8 (E-H) weeks after injection from rats treated with PBS, blank NPs, PEDF, and PEDF-NPs are shown in Figures 3 and 4. GFAP was expressed in activated Müller cells, and light immunoreactivity with the rod opsin antibody was observed in the debris between photoreceptors and the retinal pigment epithelium (RPE). However, this was similar in all preparations (Figure 3A-D). A greater number of photoreceptor nuclei remained in PEDF-NP-treated eyes than in PBS-, blank NP-, and PEDF-treated eyes (Figure 3D). At 8 weeks, subretinal gliosis of the Müller cells was observed in PBS-, blank NP- and PEDF-treated eyes, and rod opsin was missing (Figure 3E-G). In contrast, rats treated with PEDF-NPs maintained expression of rod opsin at 8 weeks (Figure 3H). Immunolabeling with the antibody to vimentin occurred throughout the Müller cells (Figure 4A-H). In PBS-, blank NP-, and PEDF-treated eyes, cone opsin was present in small amounts in the collapsed cones in the OS (Figure 4A-C), whereas expression of cone opsin was much higher in PEDF-NP-treated eyes (Figure 4D). Eight weeks after injection, cone opsin was almost missing in PBS-, blank NP-, and PEDF-treated eyes (Figure 4E-G). In contrast, rats treated with PEDF-NPs had stronger immunolabeling of cone opsin (Figure 4H). Immunostaining for PEDF was weakly observed in the RPE layer of PBS-, blank NP-, or PEDF-treated eyes (Figure 5A-C). However, the staining for PEDF was stronger in the outer retina and the RPE layer in PEDF-NP-treated eyes (Figure 5D).

### Effects of pigment epithelium-derived factor-impregnated nanoparticles on physiology

**Figure 6:**
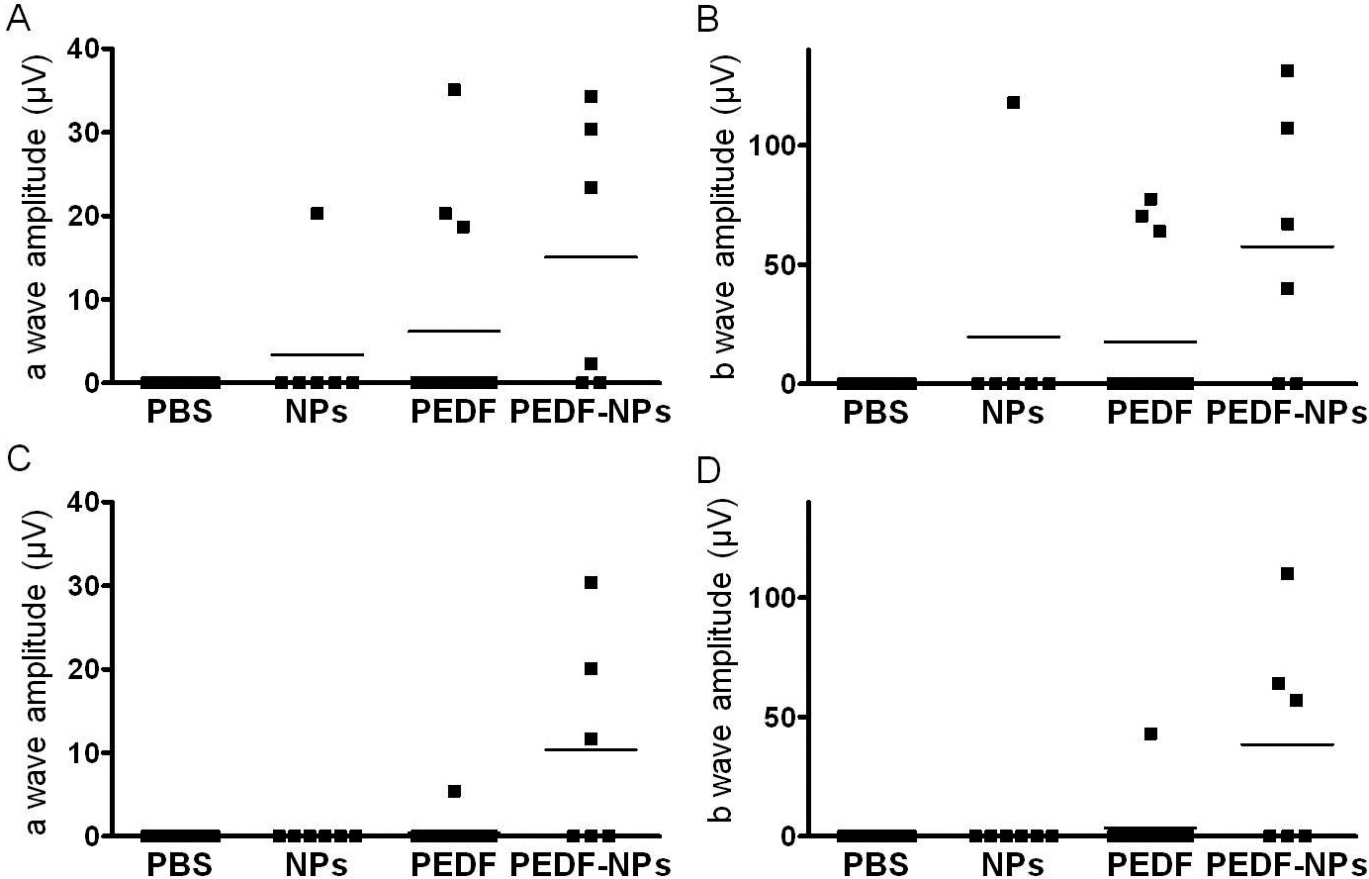
Physiologic rescue of Royal College of Surgeons (RCS) rats at 4 (**A**, **B)** and 8 (**C**, **D**) weeks after injection. The amplitudes of a- (**A**, **C**) and b- (**B**, **D**) waves were measured and plotted as each point on the graph. Each bar on the graph represents the mean amplitude for each group. PEDF-NP-treated eyes showed significant preservation of a- and b-wave amplitudes at 8 weeks after injection, compared with PEDF-treated eyes (both p=0.0339).

Four weeks after injection, the mean amplitudes of the a- and b-waves in PEDF-NP-treated eyes were larger than those in PEDF-treated eyes (Figure 6A-B). PEDF-NP-treated eyes also showed significant preservation of a- and b-wave amplitudes at 8 weeks after injection, compared with PEDF-treated eyes (both p=0.0339; Figure 6C-D).

### Effects of pigment epithelium-derived factor-impregnated nanoparticles on photoreceptor cell death

**Figure 7:**
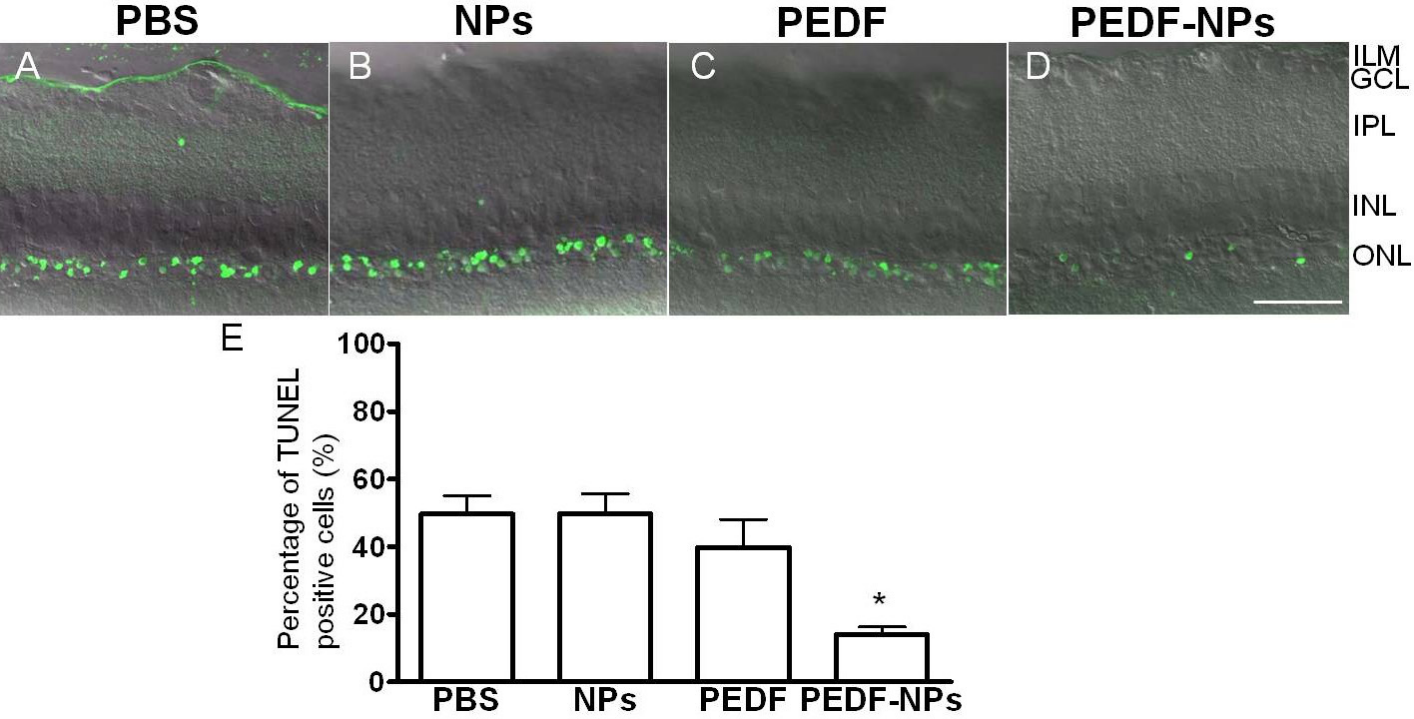
Fluorescence photomicrographs of retinal sections from a terminal deoxynucleotidyl transferase-mediated uridine 5′-triphosphate-biotin nick end labeling (TUNEL) assay at 4 weeks after injection. Representative photographs 4 weeks after injection with PBS (**A**), blank-NPs (**B**) PEDF (**C**) and PEDF-NPs (**D**) are shown. ILM; inner limiting membrane, GCL; ganglion cell layer, IPL; inner plexiform layer, INL; inner nuclear layer, ONL; outer nuclear layer. A bar represents 50 µm. **E**: Mean percentage of TUNEL-positive cells of RCS rats at 4 weeks after injection. Data are shown as the mean±SD (n=4 in each group). The percentage of TUNEL-positive cells in the ONL was significantly lower in PEDF-NP-treated eyes than in PEDF-treated eyes (*p=0.0007).

Using agarose-embedded sections, TUNEL data were collected 8 weeks after intravitreal injection of PEDF-NPs (Figure 7D) to identify TUNEL-positive cells in the ONL (Figure 7E). The average percentage of TUNEL-positive cells in the ONL in PEDF-NP-treated eyes was 14.0±2.3%, whereas in PBS-, blank NP-, and PEDF-treated eyes the average percentages were 49.6±5.5%, 49.7±5.9%, and 39.8±8.3%, respectively. The percentage of TUNEL-positive cells in the ONL was significantly lower in PEDF-NP-treated eyes than in PEDF-treated eyes (Figure 7E; p=0.0007), and was much greater in PBS- and blank NP-treated eyes (Figure 7E).

## Discussion

Intravitreal injection of PEDF has been shown to rescue rodent photoreceptors from genetic degeneration and the damaging effect of light [1,2]. In this study, our goal was to test the ability of PEDF encapsulated in gelatin NPs to rescue photoreceptors in a well established model of photoreceptor degeneration, and to determine whether NP-based delivery enhances the protective effects of PEDF.

Our previous data for the intraocular kinetics of intravitreally injected NPs show that the NPs can be delivered to photoreceptors directly and continuously [5]. This result prompted us to determine whether this system can be used as an effective drug delivery system (DDS) for PEDF for preventing photoreceptor degeneration. We found a significantly increased number of cells in the ONL and preservation of a- and b-wave amplitudes in ERG studies in PEDF-NP-treated eyes, compared to PEDF-treated eyes, at 8 weeks after injection in RCS rats. These observations suggest that intravitreal injection of PEDF-NPs delayed photoreceptor degeneration in RCS rats morphologically and functionally, and this conclusion was supported by the effect on photoreceptor apoptosis. Data collected in week 8 after injection suggested that treatment with PEDF-NPs resulted in significant morphological and functional preservation, including reduction of photoreceptor apoptosis. Taken together, the results suggest that a single intravitreal injection of PEDF-NPs has a significant survival effect on photoreceptor degeneration morphologically and functionally, through inhibition of apoptosis in RCS rat retinas due to effective targeting and sustained release of PEDF.

In the NPs, the negative charge of PEDF has an electrostatic interaction with the cationic gelatin chains. If an environmental change occurs, such as an increase in ionic strength, PEDF will be released from the PEDF-gelatin complex. Even if such a change does not occur, enzymatic degradation of the gelatin matrix itself will also lead to PEDF release, although the enzyme that degrades gelatin may also degrade PEDF. We believe that the mechanism of enzymatic degradation of the gelatin matrix is the most likely basis of PEDF release. Therefore, the present findings may suggest that PEDF is released from the NPs together with degraded gelatin fragments in the retina.

Delivery of PEDF for photoreceptor rescue has been performed by intravitreal or subretinal injection of the native protein or through introduction of the PEDF gene in a viral vector [1-4]. To maintain therapeutic levels of PEDF in vivo, subretinal injection of a viral vector encoding PEDF would be ideal; however, this method has several problems, including uncontrolled drug delivery, a difficult injection technique, limited retinal distribution, and biologic safety concerns. Our novel DDS for PEDF enables targeting to photoreceptors, controlled drug delivery in all regions of the retina, a simple injection technique, and biologic safety. In fact, we found a protective effect on photoreceptors without serious complications, suggesting that this approach for delivering PEDF will improve the therapeutic efficacy for photoreceptor degeneration without causing serious complications. Furthermore, our results indicate that a single intravitreal injection of PEDF-NPs had a longer survival effect than that of any other drugs in RCS rats [8-11]. This observation suggests that the DDS may be useful clinically in short-term photoreceptor rescue.

In conclusion, our results show that intravitreal administration of PEDF-NPs effectively delays photoreceptor degeneration in RCS rats without causing serious complications, due to the targeting and sustained release of PEDF in situ. PEDF-NPs may have advantages clinically, since the safety of gelatin has been clinically proven [12,13]. Thus, intravitreal administration of PEDF-NPs is a candidate as a new therapeutic strategy for photoreceptor degeneration.
